# Traumatic brain injury, symptoms of depression and sleep dysfunction in former elite rugby players

**DOI:** 10.1093/braincomms/fcag404

**Published:** 2026-10-27

**Authors:** Axel A S Laurell, Maria-Eleni Dounavi, Audrey Low, Elijah Mak, Maja Swirska, Graciela Muniz-Terrera, Benjamin R Underwood, Yves Dauvilliers, Ivan Koychev, Brian A Lawlor, Lorina Naci, Paresh Malhotra, William Stewart, Craig W Ritchie, John T O’Brien, PREVENT Dementia investigators, Katie Bridgeman, Leonidas Chouliaras, Siobhan Coleman, Hannah Darwin, Maria-Eleni Dounavi, Feng Deng, Robert Dudas, Sarah Gregory, Ivan Koychev, Brian Lawlor, Audrey Low, Clare Mackay, Elijah Mak, Paresh Malhotra, Graciela Muniz-Terrera, Lorina Naci, John O’Brien, Vanessa Raymont, Craig Ritchie, Li Su, Peter Swann, Tony Thayanandan, Guy Williams

**Affiliations:** Department of Psychiatry, University of Cambridge, Addenbrooke's Hospital, Cambridge CB2 2QQ, UK; Cambridgeshire and Peterborough NHS Foundation Trust, Windsor Research Unit, Fulbourn Hospital, Cambridge CB21 5EF, UK; Department of Psychiatry, University of Cambridge, Addenbrooke's Hospital, Cambridge CB2 2QQ, UK; Department of Radiology, Mayo Clinic, Rochester, MN 55905, USA; Department of Radiology, Mayo Clinic, Rochester, MN 55905, USA; Department of Psychiatry, University of Cambridge, Addenbrooke's Hospital, Cambridge CB2 2QQ, UK; Ohio University Heritage College of Osteopathic Medicine, Ohio University, Athens, OH 45701, USA; Department of Psychiatry, University of Cambridge, Addenbrooke's Hospital, Cambridge CB2 2QQ, UK; Cambridgeshire and Peterborough NHS Foundation Trust, Windsor Research Unit, Fulbourn Hospital, Cambridge CB21 5EF, UK; Sleep-Wake Disorders Center, Department of Neurology, Gui-de-Chauliac Hospital, CHU Montpellier, Université de Montpellier, Montpellier 34295, France; Department of Psychiatry, University of Oxford, Warneford Hospital, Oxford OX3 7JX, UK; Department of Brain Sciences, Imperial College London, Burlington Danes, The Hammersmith Hospital, London W12 0NN, UK; Global Brain Health Institute, Trinity College Dublin, Dublin D02 X9W9, Ireland; Global Brain Health Institute, Trinity College Dublin, Dublin D02 X9W9, Ireland; Trinity College Institute of Neuroscience, Trinity College Dublin, Dublin D02 X9W9, Ireland; Department of Brain Sciences, Imperial College London, Burlington Danes, The Hammersmith Hospital, London W12 0NN, UK; Department of Neurosurgery, Penn Center for Brain Injury and Repair, Perelman School of Medicine, University of Pennsylvania, Philadelphia, PA 19104, USA; Department of Neuropathology, Queen Elizabeth University Hospital, Glasgow G51 4TF, UK; School of Medicine, University of St Andrews, St Andrews KY16 9AJ, UK; Scottish Brain Sciences, Edinburgh EH12 9DQ, UK; Department of Psychiatry, University of Cambridge, Addenbrooke's Hospital, Cambridge CB2 2QQ, UK; Cambridgeshire and Peterborough NHS Foundation Trust, Windsor Research Unit, Fulbourn Hospital, Cambridge CB21 5EF, UK

**Keywords:** rugby, traumatic brain injury, depression, sleep, neuroimaging

## Abstract

While self-reported repetitive head impact (RHI) exposure in former athletes has been associated with symptoms of depression and sleep dysfunction, it is uncertain whether these symptoms are mediated by changes in brain structure. We used cross-sectional data from 57 middle-aged, cognitively healthy, former elite rugby players and 114 propensity-matched controls. Questionnaires were used to collect self-reported traumatic brain injury (srTBI: blows to the head; episodes of feeling dazed and confused; episodes of loss of consciousness), RHI exposure (career length, play at the highest level and playing position), medical history, symptoms of depression (Center for Epidemiological Studies Depression scale) and sleep dysfunction (Pittsburgh Sleep Quality Index). Grey matter volumes and diffusion tensor imaging measures were obtained using FreeSurfer (v7.1.0, v7.4.2) and FSL (v6.0.5). We assessed how srTBI, RHI exposure, symptoms of depression, sleep dysfunction and the imaging markers were related using Spearman correlations, multivariate regression models and partial least squares structural equation modelling. Although former rugby players reported a higher number of blows to the head (9.8 versus 5.4, *P* < 0.001), number of episodes of feeling dazed and confused (4.5 versus 1.7, *P* < 0.001) and number of episodes of loss of consciousness (1.9 versus 0.7, *P* < 0.001), they were less likely to report a past diagnosis of anxiety (7% versus 22%, *P* = 0.03) or sleep disorders (2% versus 15%, *P* = 0.02) than controls. In rugby players, srTBI correlated poorly with RHI exposure. Furthermore, srTBI, but not RHI exposure, was associated with previous depression, anxiety and sleep disorders. Higher srTBI was directly associated with more symptoms of depression [*B*: 0.52 (95%CI: 0.21–0.74)], and indirectly associated with more sleep dysfunction [*B*: 0.18 (95%CI: 0.03–0.39)], fully mediated through symptoms of depression. Higher srTBI was also associated with a larger putamen (*r* = 0.28, *P* = 0.03) and inferior-tubular hypothalamus (*r* = 0.35, *P* = 0.007), thicker medial orbitofrontal (*r* = 0.32, *P* = 0.02) and insular cortices (*r* = 0.40, *P* = 0.002), lower axial diffusivity of the inferior cerebellar peduncle (*r* = −0.28, *P* = 0.03) and uncinate fasciculus (*r* = −0.26, *P* = 0.048), lower fractional anisotropy (*r* = −0.44, *P* < 0.001) and higher radial diffusivity (*r* = 0.29, *P* = 0.03) of the tapetum. Symptoms of depression were associated with thicker superior (*r* = 0.30, *P* = 0.02) and inferior (*r* = 0.33, *P* = 0.01) parietal cortices, higher fractional anisotropy of the hippocampal cingulum (*r* = 0.27, *P* = 0.04) and lower mean diffusivity of the fornix (*r* = −0.28, *P* = 0.03). None of these imaging markers mediated the association between srTBI and symptoms of depression. SrTBI, but not RHI exposure, was associated with symptoms of depression in former rugby players. However, this relationship was not mediated through differences in brain structure, suggesting that psychosocial or methodological factors such as recall bias and common method bias may be more important.

## Teaser

Laurell *et al.* found that self-reported, but not objective, head injury burden was associated with depression in former elite rugby players. This was not mediated through differences in brain structure and they conclude that the association may be due to psychosocial or methodological factors rather than brain injury.

## Graphical Abstract

**Graphical Abstract:**
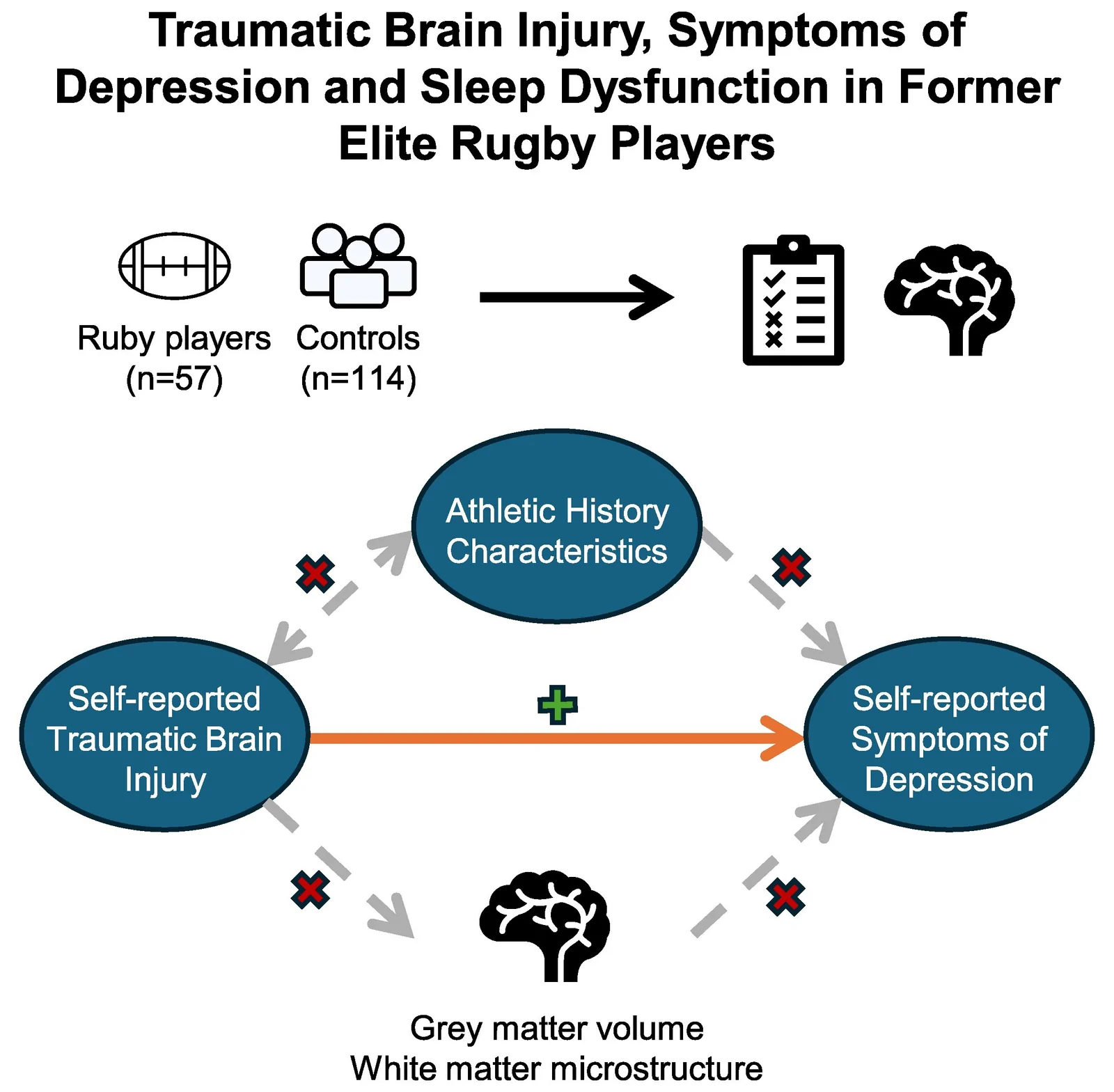

## Introduction

Contact sports, such as American football and rugby, are associated with frequent repetitive head impact (RHI) exposure, as well as episodes of traumatic brain injury (TBI) with concussive symptoms. Both RHI exposure and TBI are recognized as potentially modifiable risk factors for neurodegenerative disease among former elite level participants of contact sports.^1^ Associated with this is the frequent finding of a neuropathology specific to prior TBI and RHI exposure, chronic traumatic encephalopathy neuropathologic change (CTE-NC), in autopsy examinations of former contact sports athletes.^2^ To date, the clinical correlates of CTE-NC remain incompletely understood, and are described in research consensus criteria as the traumatic encephalopathy syndrome (TES).^3^ These propose evaluation of possible TES based on core criteria, while also listing various supportive clinical features as ‘frequently present’ but lacking sufficient evidence of specificity to be considered core diagnostic criteria. Among these, psychiatric symptoms, including depression, are suggested as common among individuals with autopsy-proven CTE-NC. However, given the prevalence of psychiatric disorders in the general population, it remains uncertain whether anxiety, depressive symptoms and poor sleep are more frequent in former athletes and whether they are associated with evidence of underlying structural changes in the brain.

Studies of people attending hospital after a TBI report a high prevalence of anxiety, depression and sleep dysfunction (including insomnia, hypersomnia and parasomnias) persisting years after the injury in some individuals.^4-6^ With respect to sports-associated RHI exposure and TBI, a higher number of self-reported concussions are associated with an increased risk of depression among former professional American football players.^7-9^ In contrast, the evidence from studies of retired rugby players is more mixed, with some studies reporting that ex-players have high rates of anxiety, depression and sleep dysfunction, with these symptoms correlating with self-reported concussion exposure.^10-12^ Other studies report that while retired rugby players might have subtle cognitive differences,^13,14^ there was no observed increase in anxiety and depression compared to age-, sex- and deprivation-matched healthy controls.^13^ Notably, most previous studies have only considered self-reported depression, sleep dysfunction and previous concussions, without corroborating findings with more objective proxies of RHI exposure (such as career length or player position) or brain imaging. As such, they are prone to recall bias, especially as the potential neuropsychiatric sequelae of RHI exposure and TBI have received considerable media coverage.

Abnormal brain imaging following TBI is a risk factor for subsequent depression in the hospital clinic population, but the anatomical locations of lesions have been inconsistent.^4,15^ Furthermore, connectivity studies using functional MRI have found that depression after TBI is associated both with increased and with reduced connectivity in areas involved in emotional processing such as the amygdala, insula and anterior cingulate cortex.^16-18^ Although there are few studies examining the mechanisms by which RHI exposure and TBI might contribute to sleep dysfunction, it could be mediated by damage to the brain areas involved in sleep homeostasis, such as the hypothalamus.^19^ We have previously found that the hypothalamus is affected in people with AD,^20^ and in cognitively healthy apolipoprotein E4 (APOE4) homozygotes,^21^ and that lower hypothalamic volume is associated with sleep dysfunction.^21^ Furthermore, previous studies have found that rugby players have differences in white matter structure, and lower hippocampal and amygdala volumes,^12,22,23^ which are brain regions in close proximity to the hypothalamus.

Our aim in this study was to assess whether previous TBI and RHI exposure, measured using both self-report and athletic history characteristics, were associated with symptoms of depression and sleep dysfunction (including self-reported sleep quality, insomnia and hypersomnia) in a cohort of well-characterized, former professional rugby players. Based on previous research, we hypothesized that former professional rugby players would report more anxiety, depression and sleep dysfunction and have lower hypothalamic volume when assessed against closely matched non-rugby players. We also hypothesized that the association between TBI history and depressive symptoms would be more pronounced in former rugby players than in controls. Finally, we hypothesized that depression and sleep dysfunction after RHI exposure and TBI would be associated with differences in brain structure, particularly in the hypothalamus due to its role in sleep homeostasis. To achieve this, we leveraged the comprehensive dataset acquired through the established PREVENT dementia programme.

## Materials and methods

### Participants

The full protocol for the PREVENT Dementia cohort study has been previously described.^24,25^ Seven hundred cognitively healthy participants aged 40–59 were recruited from five sites in the UK and Republic of Ireland (Cambridge, Oxford, London, Edinburgh, Dublin). In addition to this core cohort, 60 former elite rugby union players were recruited and assessed at the Edinburgh study site using the same protocol as had been used for the core PREVENT cohort. One participant who had been recruited as part of the main PREVENT cohort was a former professional rugby player, and was therefore included in the rugby group. Of the 61 former elite rugby players in this study, 58 reported that they had played to national representative level.

### Consent statement

All human subjects included in this study provided informed consent.

### Ethical approval

The PREVENT dementia programme received multi-site ethical approval from the UK London-Camberwell St Giles National Health Service (NHS) Research Ethics Committee (REC reference: 12/LO/1023, IRAS project ID: 88938), which operates according to the 1975 Declaration of Helsinki (and as revised in 1983). Separate ethical approval for Ireland was obtained from the Trinity College Dublin School of Psychology Research Ethics Committee (SPREC022021-010) and the St James Hospital/Tallaght University Hospital Joint Research Ethics Committee.

### Traumatic brain injury measures

Previous history of TBI was assessed using the self-reported Brain Injury Screening Questionnaire (BISQ) which was administered to all participants, asking them to recall head injuries in 20 specific scenarios.^26^ For each scenario, the BISQ asks participants about the number of blows to the head, episodes of feeling dazed and confused and episodes of loss of consciousness following a head injury. Participants are also asked to report the longest period of being either dazed and confused or unconscious following a head injury. Mild TBI was identified as an episode of loss of consciousness with a duration of less than 30 min.^27^ Self-reported TBI burden (srTBI) was assessed with three variables: the lifetime number of blows to the head (nBL), the lifetime number of times a head injury resulted in feeling dazed and confused (nDC) and the lifetime number of times a head injury resulted in loss of consciousness (nLC). The srTBI measures were treated as continuous variables, but we also performed an exploratory analysis by binarizing them into ‘high’ and ‘low’ exposure groups based on the median (Supplementary Results). In the rugby player cohort, we collected three athletic history characteristics, as proxies of RHI exposure: total career length; play at the highest level (length of time × frequency playing at the highest level) and playing position dichotomized as forwards (flanker, number eight, prop, hooker) or backs (centre, scrum-half, fly-half, full-back, wing) reflecting higher versus lower head impact risk positions.^28^

### Alcohol

Participants were asked about how many glasses of wine, beer and ‘stronger alcohol’ they drank per week. The number of units per week was calculated by assuming 2 units per glass of wine and beer, and 1 unit per glass of stronger alcohol.

### Physical activity

Participants were asked to rate on a 6-point ordinal scale how often they engaged in mild, moderate and vigorous sports or physical activities (0 = never, 1 = less than monthly, 2 = monthly, 3 = fortnightly, 4 = weekly, 5 = daily).

### Medical, psychiatric and sleep comorbidities

Participants were asked in a structured interview whether they had been diagnosed with any medical or psychiatric conditions, including depression, anxiety and sleep disorders. In addition, ongoing symptoms of depression were assessed using the Center for Epidemiological Studies Depression (CESD) scale in which participants were asked about the frequency of 20 symptoms of depression during the past week.^29^ This results in a score out of 60, with higher scores indicating more frequent symptoms of depression.

Subjective symptoms of sleep dysfunction were assessed using the Pittsburgh Sleep Quality Index (PSQI).^30^ This provides a measurement for sleep quality (PSQI component 1), latency (PSQI component 2), duration (PSQI component 3), efficiency (PSQI component 4), disturbances (PSQI component 5), use of sleeping medications (PSQI component 6) and daytime dysfunction (PSQI component 7), with each component being scored on a 4-point ordinal scale which are then combined to give a global score between 0 and 21. The PSQI was developed and validated as a screening instrument to distinguish between ‘good’ and ‘poor’ sleepers by using the cutoff of > 5 on the PSQI global score. Scoring above the cutoff on the PSQI global score would usually indicate moderate–severe difficulties (scoring ≥ 2 on a PSQI component) in two to three different areas of sleep.

Several additional questionnaires were used to assess the risk of common causes of sleep dysfunction. These were available for the full cohort of ex-rugby players and half of the main PREVENT cohort due to their inclusion in the study protocol after the study start. Excessive daytime sleepiness was defined as scoring >10 out of 24 on the Epworth Sleepiness Scale.^31^ We also used the NoSAS (Neck circumference, Obesity, Snoring, Age and Sex) score, which is a screening tool used to identify individuals at high risk of sleep disordered breathing.^32^ The cutoff of ≥ 8/17 has a positive predictive value of 33–47% and negative predictive value of 90–98% for sleep disordered breathing indicating its value as a ‘rule-out’ screening tool. Suspected rapid eye movement sleep behaviour disorder (RBD) was assessed using a single screening question, which has been previously shown to have a high sensitivity and specificity.^33^ Finally, participants were considered to have suspected restless legs syndrome (RLS) if they endorsed four questions relating to the diagnostic criteria of the disorder, and confirmed that their symptoms were not attributable to another condition.^34^

### Image acquisition and analysis

The PREVENT-Dementia protocol included a T1-weighted magnetization-prepared rapid gradient echo (MPRAGE) sequence (repetition time = 2.3 s, echo time = 2.98 ms, 160 slices, flip angle = 9 degrees, voxel size = 1 mm^3^ isotropic) using the following 3T scanners: Siemens Prisma (Oxford, Edinburgh), Prisma fit (Cambridge), Verio (West London, Edinburgh) and Skyra (Dublin and Edinburgh). Four participants from the rugby sub-study were excluded due to incomplete imaging sequences. All scans were corrected for field inhomogeneities using the Advanced Normalization Toolbox (ANTs) N4 algorithm.^35^

#### Grey matter structure

All T1-weighted images were quality-controlled, following correction with N4 for bias field inhomogeneities. Subsequently, the FreeSurfer (version 7.1) standard recon-all pipeline was run on the dataset to obtain 87 regional grey matter volumes (GMVs).^36^ Right and left-sided structures were averaged to obtain 45 GMVs covering the volume and thickness of cortical and subcortical structures. Hypothalamic volume and the volume of five hypothalamic subunits (anterior–superior, anterior–inferior, superior–tubular, inferior–tubular, posterior), were obtained by running an additional module in FreeSurfer (version 7.4.2), and the volume of the right and left hypothalamic subunits were then combined for the analysis.^37^ All images and segmentations were manually checked for quality control, done blind to knowledge of whether the participant was a rugby player or control.

#### White matter microstructure

Diffusion tensor imaging (DTI) data were pre-processed and analysed using FSL (version 6.0.4). First, the first volume of the DTI series was extracted to serve as the reference b0 image. Brain masks were generated from the b0 image and the mask generated with the fractional intensity threshold of 0.2 used for subsequent processing steps. Following correction for eddy currents and subject motion,^38^ the diffusion tensor model was fitted to the data to generate maps of fractional anisotropy (FA), mean diffusivity (MD), radial diffusivity (RD) and axial diffusivity (AxD). We registered the Johns Hopkins University-International Consortium for Brain Mapping (JHU-ICBM) white matter atlas to each subject’s native space using the brain mask, derived from FreeSurfer.^39^ The JHU-ICBM white matter labels atlas was then transformed to each subject’s native space using the inverse of the previously computed transformation.^40^ Finally, FA, MD, RD and AxD values were calculated across 50 white matter tract regions of interest (ROIs). Right- and left-sided structures were averaged to create 26 separate white matter ROIs.

#### Harmonization of imaging data

We used NeuroCombat harmonization to correct the hypothalamic volume and subunit volumes for the effects of using different MRI coils and scanner models at the different recruitment sites.^41,42^ Modulators in the harmonization were age, sex, years of education, family history of dementia (FHD), apolipoprotein ε4 (APOE4) genotype and whether the participant was a rugby player or a PREVENT control. The remaining GMVs and DTI measures were only used for analysing data from the rugby group. Since imaging data for this group were all acquired using the same MRI scanner, harmonization was not required.

### Statistical analysis

#### Propensity score matching

Since rugby players are likely to have many characteristics which are different compared to the population included in the core PREVENT cohort, we conducted propensity score matching at a 2:1 ratio, whereby two PREVENT cohort participants with complete imaging sequences were matched to each rugby participant. Propensity matching was based on age, sex, years of education, body mass index (BMI), smoking status, dementia family history, APOE4 genotype, level of physical activity and systolic blood pressure at the baseline assessment. Prior to matching, missing data were imputed using Multiple Imputation by Chained Equations (MICE). For all variables, < 5% of the data were missing, with no more than two missing data points in any one participant. Participants were matched by propensity score using the nearest neighbour method, with callipers set at 0.2.^43^ Propensity matching and missing data imputation were performed in R version 4.0.3. MICE was also used to impute missing values in some of the questionnaire data, including the PSQI, CESD, alcohol, NoSAS and RBD, all which had < 5% missing data.

#### Group comparisons

All statistical comparisons were performed in R Studio, version 2024.12.1. Group differences in the demographic variables, depressive symptoms and sleep dysfunction were assessed using the Mann–Whitney U test for continuous data, and chi-square test for categorical data. We used spearman correlations and the Mann–Whitney U test, where appropriate, to assess whether the srTBI variables were significantly associated with proxies of RHI exposure.

#### Hypothalamic volume

A series of robust linear regression models, adjusted for age, sex and total intracranial volume (TIV), were used to assess whether there were any group differences in hypothalamic volume or subunit volume, and whether srTBI or proxies of RHI exposure were associated with volumes within the rugby group. We corrected for false discovery rate (FDR) for each comparison across the three measures of srTBI and across the three proxy measures of RHI exposure.

#### Associations between TBI, depression and sleep

Within the rugby group, we used a series of robust linear and logistic regression models, adjusted for age and sex, to assess whether srTBI or proxies of RHI exposure were associated with depressive symptoms, sleep dysfunction (PSQI total), proxy measures of sleep disorders (Epworth total, NoSAS total, RLS and RBD) and a past diagnosis of depression, anxiety or sleep dysfunction. Since past diagnosis of depression, anxiety or sleep dysfunction were highly correlated, these were combined into one binary variable. Due to the right-skewed distribution of the ordinal PSQI component scores, it was not possible to use linear regression models, so simple spearman correlations were used to analyse these variables. We explored whether there were any group differences in the strength of the association between srTBI measures and symptoms of depression by including an interaction term in the models [(srTBI measure)*Group]. FDR correction was applied for each comparison across the three measures of srTBI and across the three proxies of RHI exposure.

#### Structural equational modelling (SEM)

The *seminr* package in R was used to perform two sets of mediation analyses using partial least squares (PLS) SEM. The variable selection was based on the previous results from the simple correlations and multivariate regression models. Firstly, we assessed whether there was an indirect association between srTBI (nBL and nLC) and sleep dysfunction, mediated through increased symptoms of depression. Secondly, we used PLS-SEM to assess whether the association between srTBI (nBL and nLC) and symptoms of depression was mediated through differences in grey or white matter structures. The anatomical location of lesions contributing to depression following TBI is poorly understood, and previous neuroimaging studies have been inconsistent.^4,15-18^ We therefore opted for a data-driven approach to select which grey and white matter variables to examine as mediators. Firstly, a series of spearman correlations, uncorrected for multiple comparisons, were used to identify imaging markers which were significantly associated with either srTBI or symptoms of depression. Only the significant imaging variables were then entered in the SEM model. A linear model was used to regress out the effect of age and sex from all factors prior to running SEM. In addition, we regressed out the effect of TIV from all imaging measures.

#### Power calculation

In our cohort of 57 former rugby players, we would have 80% power to detect statistically significant correlations (α = 0.05, one-sided), provided that the effect size was at least moderate in magnitude (*r* ≥ 0.32).

## Results

Complete MRI imaging and hypothalamic segmentation data were available for 57 former rugby players who were then propensity matched with 114 controls. Former rugby players reported a higher nBL (9.8 versus 5.4, *P* < 0.001), a higher nDC (4.5 versus 1.7, *P* < 0.001), and a higher nLC (1.9 versus 0.7, *P* < 0.001) compared to their matched controls (Table 1). As expected, this was driven by differences in the number of sports-related (BISQ question 15) nBL (6.7 versus 1.2, *P* < 0.001), nLC (1.3 versus 0.1, *P* < 0.001) and nDC (3.4 versus 0.3, *P* < 0.001), whereas there were no significant group differences in non-sports-related nBL (3.1 versus 4.2, *P* = 0.29), nLC (0.6 versus 0.7, *P* = 0.79) or nDC (1.1 versus 1.4, *P* = 0.48). Among the participants reporting at least one episode of feeling dazed and confused (89% of rugby players, 53% of controls), or at least one episode of loss of consciousness (58% of rugby players, 32% of controls), rugby players were more likely to have been hospitalized at least once as a result of a concussion (46% versus 20%, *P* = 0.003). However, there were no significant group differences in the longest period of being either dazed and confused (*P* = 0.07) or unconscious (*P* = 0.32) as a result of a head injury. Two of the controls and none of the former rugby players reported at least one episode of moderate/severe TBI, and all other episodes of TBI were classified as mild. The former rugby players reported a mean 22 (range: 7–46) years of play, with 8 (range: 1–19) years of play at the highest level, and with 47% playing as forwards. Forwards reported a higher nBL (11.7 versus 7.7, *P*(FDR) = 0.04) and nDC (6.1 versus 3.2, *P*(FDR) = 0.02), but not nLC (2.0 versus 1.9, *P*(FDR) = 0.80) compared with backs. There were no significant associations between the other proxies of RHI exposure and srTBI measures.

**Table 1:** Rugby players reported significantly more blows to the head, more episodes of feeling dazed and confused, more episodes of loss of consciousness

	Rugby players	Controls	*P*
*n*	57	114	
Age	48.5	48.4	0.99
Sex (%women)	44	46	0.87
Body mass index	29.0 (3.5)	28.4 (6.3)	0.1
Alcohol (units/week)	9.4 (9.1)	13.0 (12.6)	0.14
Number of blows to the head	9.8 (5.3)	5.4 (6.5)	<0.001
Number of times feeling dazed and confused	4.5 (3.8)	1.7 (2.7)	<0.001
Number of losses of consciousness	1.9 (2.4)	0.7 (1.1)	<0.001
Mild physical activity	4.7 (0.7)	4.8 (0.4)	0.78
Moderate physical activity	4.6 (0.7)	4.2 (0.9)	0.002
Vigorous physical activity	4.2 (1.0)	3.8 (1.1)	0.002
Medical history of depression (%)	11	23	0.08
Medical history of a sleep disorder (%)	2	15	0.02
Medical history of an anxiety disorder (%)	7	22	0.03
Symptoms of depression (CESD total)	8.0 (7.2)	9.1 (9.1)	0.65
Sleep dysfunction (PSQI global score)	5.6 (3.1)	6.4 (3.9)	0.35
Subjective sleep quality (PSQI component 1)	1.1 (0.8)	1.1 (0.9)	0.91
Sleep latency (PSQI component 2)	0.8 (1.0)	1.0 (0.9)	0.21
Sleep duration (PSQI component 3)	0.8 (0.9)	1.0 (0.9)	0.06
Habitual sleep efficiency (PSQI component 4)	0.6 (0.8)	0.7 (1.0)	0.82
Sleep disturbances (PSQI component 5)	1.3 (0.5)	1.3 (0.6)	0.86
Use of sleeping medications (PSQI component 6)	0.2 (0.5)	0.3 (0.7)	0.47
Daytime dysfunction (PSQI component 7)	0.8 (0.7)	0.7 (0.7)	0.45
‘Poor’ sleepers (%)	44	47	0.79

They also endorsed more frequent moderate and vigorous physical activity and were less likely to report a history of a sleep disorder or anxiety disorder [values are mean (standard deviation), unless otherwise stated]. Group differences were assessed using the Mann–Whitney U test for continuous data, and chi-square test for categorical data. ‘Poor’ sleepers scored > 5 on the PSQI global score. CESD: Center for Epidemiological Studies Depression scale; PSQI: Pittsburgh Sleep Quality Index.

As a result of the propensity-matching, there were no group differences in age, sex, BMI or mild physical activity, although former rugby players still reported more frequent moderate and vigorous physical activity. There were also no differences in ongoing symptoms of depression (CESD score), sleep dysfunction (PSQI total score), PSQI component scores or alcohol consumption. The prevalence of lifetime history of diagnosed sleep disorder (2% versus 15%, *P* = 0.02) and anxiety disorder (7% versus 22%, *P* = 0.03) were lower among former rugby players than controls, with a trend towards a lower frequency of depression (11% versus 23%, *P* = 0.08). All ex-rugby players (*n* = 57) and a subgroup of the controls (*n* = 52) completed the additional sleep questionnaires. There were no group differences in excessive daytime sleepiness, risk of sleep disordered breathing, suspected REM sleep behaviour disorder or restless leg syndrome (Table 2).

**Table 2:** There was no difference between rugby players and controls in their risk of common sleep disorders

	Rugby players	Controls	*P*
*n*	57	52	
Epworth total	6.81 (3.98)	6.54 (5.12)	0.32
Excessive daytime sleepiness (%)	14	15	1
Snoring (%)	39	42	0.84
Neck circumference (cm)	38.8 (4.4)	37.8 (3.9)	0.13
NoSAS total	7.65 (4.2)	7.17 (4.5)	0.52
Suspected sleep disordered breathing (%)	42%	38%	0.85
Suspected RBD (%)	18	19	1
Suspected RLS (%)	19	13	0.57

Group differences were assessed using the Mann–Whitney U test for continuous data, and chi-square test for categorical data. RBD: rapid eye movement sleep behaviour disorder, RLS: restless leg syndrome.

### TBI and hypothalamic volume

There were no group differences in total hypothalamic volume or in any hypothalamic subunits. However, among former rugby players, play at the highest level (length × frequency) was associated with lower volume of the anterior-superior hypothalamus [*B* = −0.23 (SE: 0.07), *P*(FDR) = 0.004, *n* = 49], even adjusting for the effect of age, sex and TIV (Fig. 1A). Furthermore, forwards had a larger anterior-superior hypothalamus compared with backs [*B* = 4.79 (SE: 2.20) mm^3^, *P*(FDR) = 0.04, *n* = 48] (Fig. 1B). Since the forwards reported less play at the highest level, we repeated the model with both variables as predictors. This revealed that only play at the highest level, and not playing position, was significantly associated with lower volume of the anterior-superior hypothalamus. There was no significant association between srTBI, or total career length, and hypothalamic volume.

**Figure 1:**
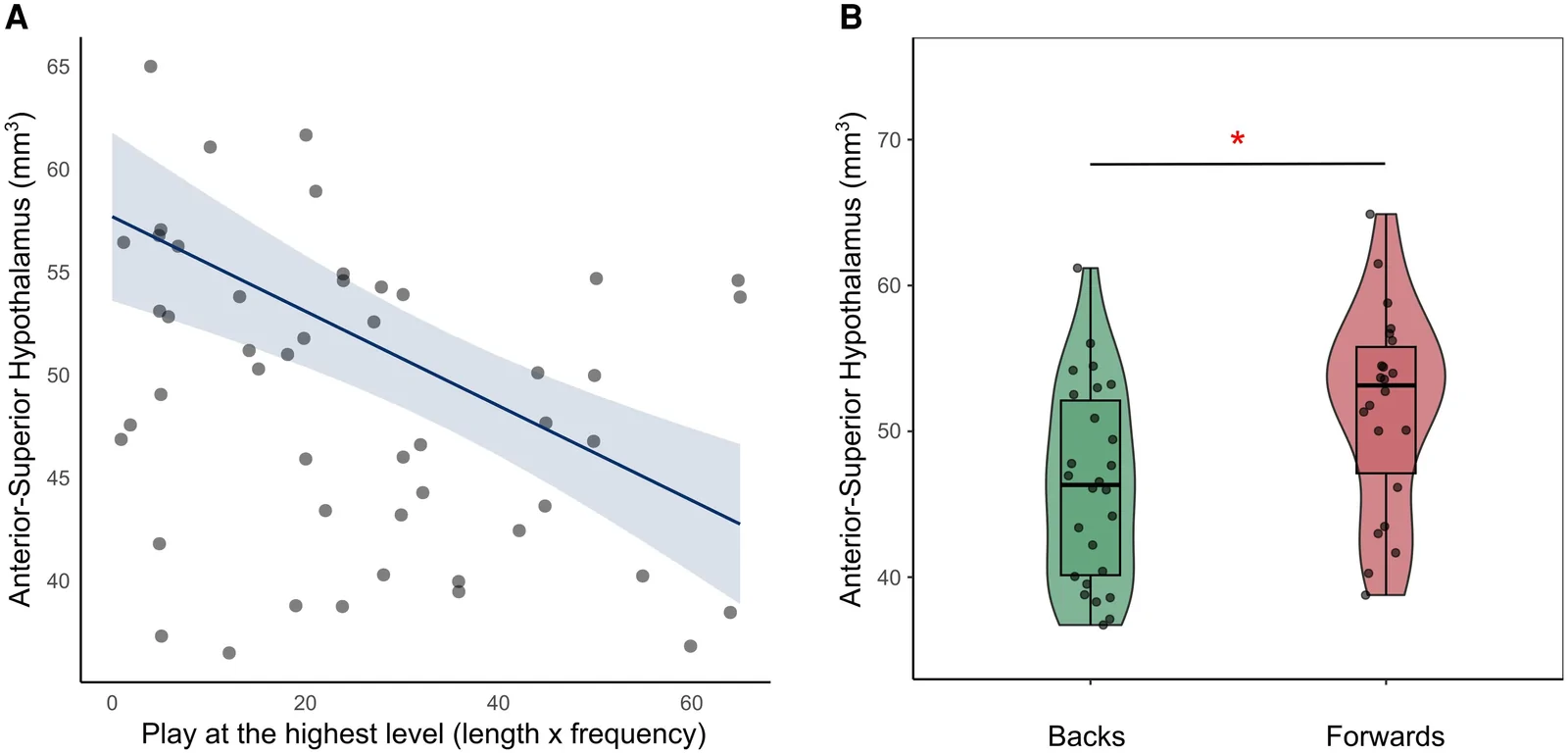
**Athletic history characteristics and hypothalamic volume.** (**A**) Within the rugby group, play at the highest level (length × frequency) was associated with a smaller anterior-superior hypothalamus [*B* = −0.23 (SE: 0.07), *P*(FDR) = 0.004, *n* = 49]. (**B**) Forwards had a larger anterior-superior hypothalamus compared with backs [*B* = 4.79 (SE: 2.20) mm^3^, *P*(FDR) = 0.04, *n* = 48]. Results were obtained using robust linear regression, adjusting for the effect of age, sex and total intracranial volume.

### TBI, symptoms of depression and sleep dysfunction

In former rugby players, a higher nBL [*B* = 0.44 (SE: 0.12), *P*(FDR) = 0.001], nLC [*B* = 1.16 (SE: 0.27), *P*(FDR) < 0.001], but not nDC [*B* = 0.35 (SE: 0.18), *P*(FDR) = 0.06] were associated with more symptoms of depression (CESD total). Similarly, a higher nDC [*B* = 0.29 (SE: 0.12), *P*(FDR) = 0.044] and nLC [*B* = 0.38 (SE: 0.18), *P*(FDR) = 0.047], but not nBL [*B* = 0.09 (SE: 0.08), *P*(FDR) = 0.28], were significantly associated with a higher prevalence of lifetime diagnosis of depression, anxiety or sleep disorder (combined variable). The association between nBL and symptoms of depression was stronger in former rugby players than in controls [nBL*Group: *B* = 0.40 (SE: 0.17), *P*(FDR) = 0.03; Fig. 2A], while the association between nDC and symptoms of depression was weaker in former rugby players than in controls [nDC*Group: *B* = −0.82 (SE: 0.29), *P*(FDR) = 0.02; Fig. 2B]. There were no significant group differences in the association between nLC and symptoms of depression [nLC*Group: *B* = 0.26 (SE: 0.62), *P*(FDR) = 0.67; Fig. 2C], and the relationships between the srTBI measures and lifetime diagnosis of depression, anxiety or sleep disorder.

**Figure 2:**
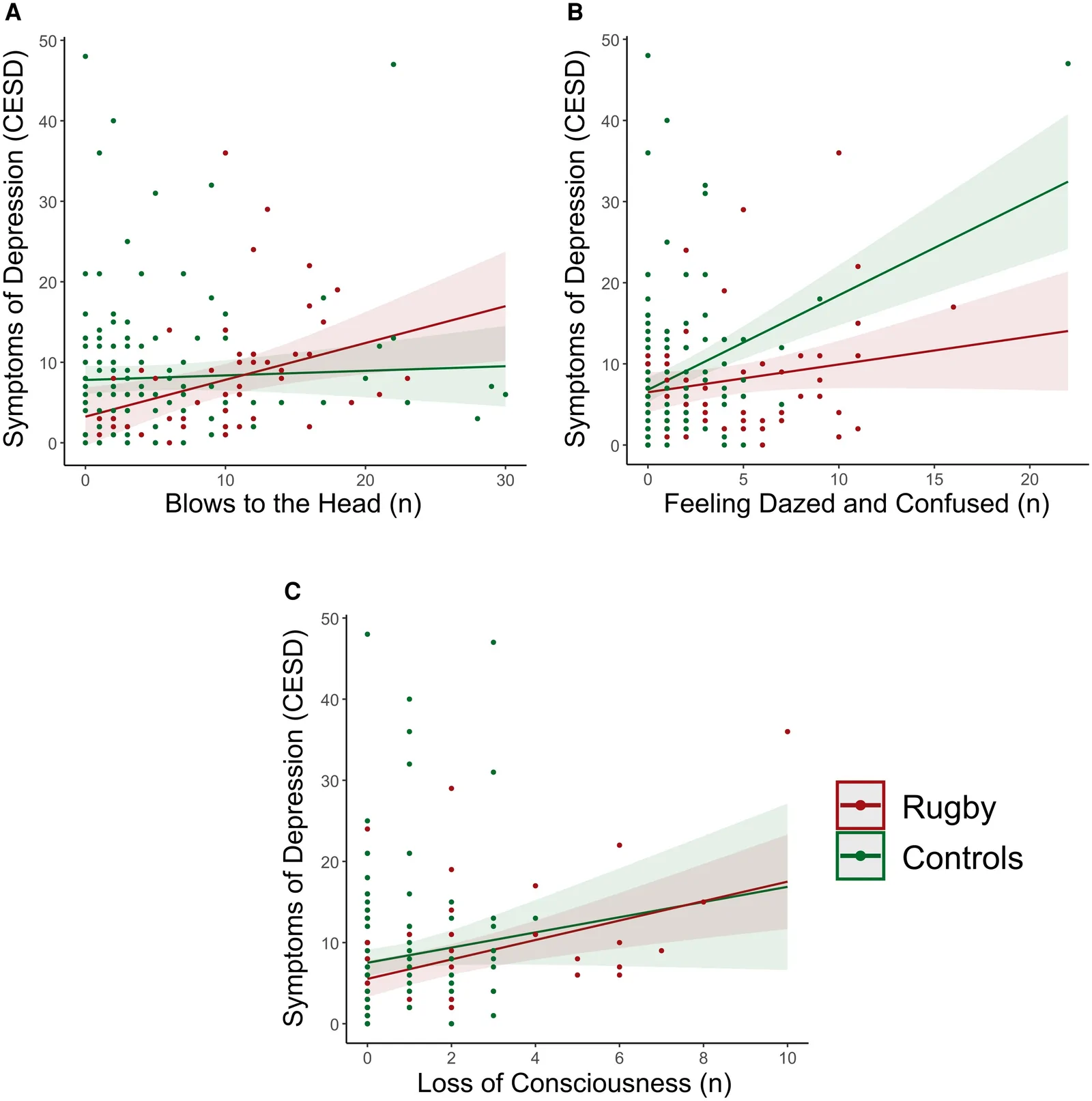
**Self-reported traumatic brain injury is associated with symptoms of depression.** The association between the lifetime number of blows to the head (nBL) and symptoms of depression was stronger in former rugby players than in controls [nBL*Group: *B* = 0.40 (SE: 0.17), *P*(FDR) = 0.03, **A**], while the association between the lifetime number of episodes of feeling dazed and confused (nDC) and symptoms of depression was weaker in former rugby players than in controls [nDC*Group: *B* = −0.82 (SE: 0.29), *P*(FDR) = 0.02, **B**]. There were no significant group differences in the association between lifetime number of episodes of loss of consciousness (nLC) and symptoms of depression [nLC*Group: *B* = 0.26 (SE: 0.62), *P*(FDR) = 0.67, **C**]. Results were obtained using robust linear regression, adjusting for the effect of age and sex (*n* = 171). CESD: Center for Epidemiological Studies Depression scale.

There were no significant associations between the proxies of RHI exposure and symptoms of depression, or past diagnosis of depression, anxiety or sleep disorder. Neither srTBI nor proxies of RHI exposure were associated with any measure of sleep dysfunction or any of the proxy measures of sleep disorders, except for a trend-level association between a higher nDC and a higher risk of sleep disordered breathing [NoSAS total, *B* = 0.24 (SE: 0.10), *P* = 0.02, *P*(FDR) = 0.07]. The results were very similar when repeating the analysis using binarized rather than continuous measures of srTBI (Supplementary Results).

### Mediation analysis with depression

Among former rugby players, PLS-SEM demonstrated a strong direct association between nLC and symptoms of depression [*B*: 0.52 (95%CI: 0.21–0.74)], and a moderate indirect association between nLC and sleep dysfunction, mediated through symptoms of depression [*B*: 0.18 (95%CI: 0.03–0.39)] (Fig. 3). There was no significant direct association between nLC and sleep dysfunction [*B*: 0.0 (95%CI: −0.21–0.21)], suggesting full mediation through symptoms of depression. Repeating the model using the nBL rather than nLC variable produced very similar results (Supplementary Fig. 1).

**Figure 3:**
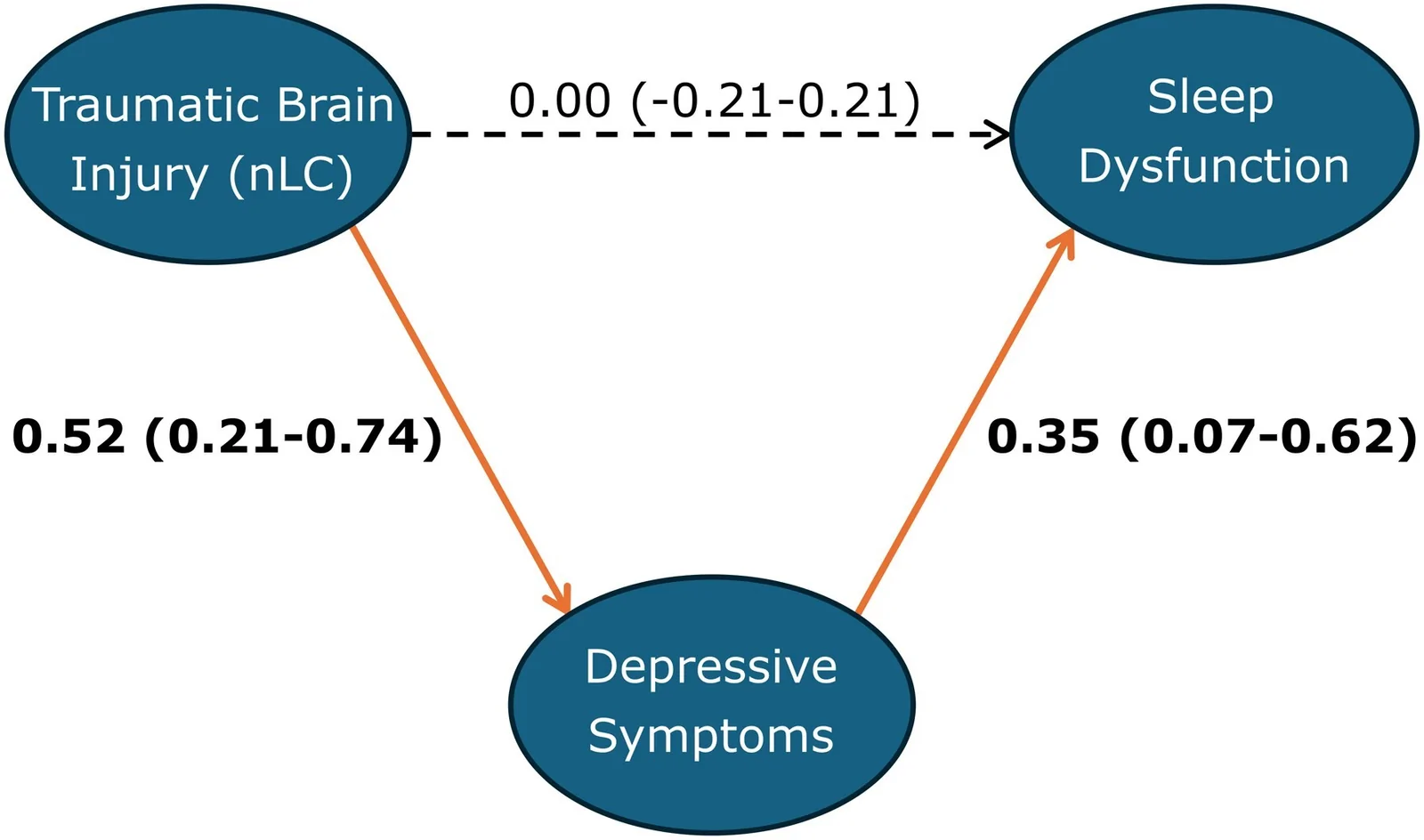
**The association between traumatic brain injury and sleep dysfunction is mediated by symptoms of depression.** There was a strong direct association between lifetime number of episodes of loss of consciousness and symptoms of depression (Center for Epidemiological Studies Depression scale), and a moderate indirect association between the number of episodes of loss of consciousness and sleep dysfunction (Pittsburgh Sleep Quality Index), mediated through symptoms of depression. These results were obtained using partial least squares structural equation modelling (*n* = 57).

### Mediation analysis with brain structure

We used PLS-SEM to determine whether the association between srTBI and symptoms of depression was mediated through differences in brain structure. The brain imaging variables included in the model were selected through a series of spearman correlations uncorrected for multiple comparisons, assessing which variables were associated with either srTBI or symptoms of depression. Higher nLC was associated with a larger putamen (*r* = 0.28, *P* = 0.03), larger inferior tubular hypothalamus (*r* = 0.35, *P* = 0.007), higher thickness of the medial orbitofrontal cortex (*r* = 0.32, *P* = 0.02), higher thickness of the insular cortex (*r* = 0.40, *P* = 0.002) and lower AxD of the inferior cerebellar peduncle (*r* = −0.28, *P* = 0.03). Higher nBL was associated with lower FA (*r* = −0.44, *P* < 0.001) and higher RD (*r* = 0.29, *P* = 0.03) of the tapetum, and lower AxD of the uncinate fasciculus (*r* = −0.26, *P* = 0.048). More symptoms of depression were associated with greater thickness of the superior (*r* = 0.30, *P* = 0.02) and inferior parietal cortices (*r* = 0.33, *P* = 0.01), higher FA of the hippocampal cingulum (*r* = 0.27, *P* = 0.04) and lower MD of the fornix (*r* = −0.28, *P* = 0.03). PLS-SEM demonstrated that none of these imaging variables mediated the association between nLC and symptoms of depression (Fig. 4), or between nBL and symptoms of depression (Supplementary Fig. 2).

**Figure 4:**
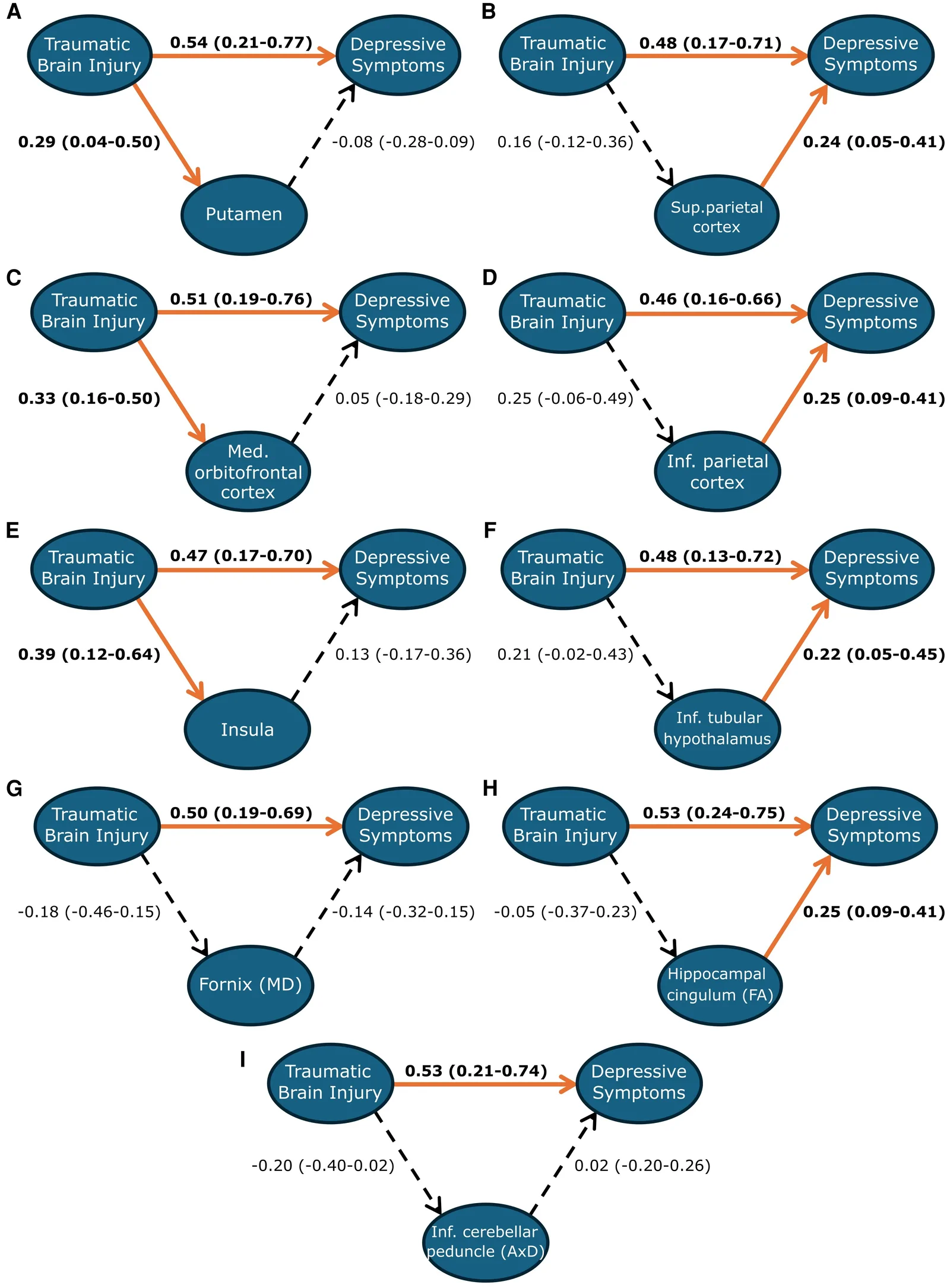
**The association between traumatic brain injury and symptoms of depression is not mediated by grey or white matter structures.** There were nine brain regions (panels **A–I**) which were significantly associated with either self-reported traumatic brain injury (srTBI, number of episodes of loss of consciousness) or symptoms of depression. However, none of these mediated the association between srTBI and symptoms of depression. Solid lines denote significant associations while dashed lines denote the non-significant associations between factors. These results were obtained using partial least squares structural equation modelling (*n* = 57). AxD: axial diffusivity; FA: fractional anisotropy; Inf: inferior; MD: mean diffusivity; Med: medial; Sup: superior.

## Discussion

In this study we assessed whether history of participation in contact sport with associated high exposure to RHI and TBI was associated with symptoms of depression, sleep dysfunction and brain structure in a cohort of former elite rugby players and their matched controls. While the former rugby players were less likely to report a previous diagnosis of anxiety or sleep disorders than controls, we found that srTBI was strongly associated with self-reported symptoms of depression and with a past diagnosis of depression, anxiety and sleep disorder. There was also an indirect association between srTBI and sleep dysfunction, mediated through symptoms of depression. However, objective proxies of RHI exposure were not associated with symptoms of depression and correlated poorly with srTBI. In addition, we did not find that the association between srTBI and symptoms of depression was mediated by grey or white matter differences. Indeed, both greater srTBI and symptoms of depression were mostly associated with higher GMV and less microstructural white matter changes. These associations are in the opposite direction to what would be expected if differences in brain structure were driving the association with symptoms of depression. This suggests that the strong association between srTBI and self-reported depressive symptoms in former elite rugby players may be due to psychosocial or methodological factors, other than damage to brain structures. For example, former rugby players who are experiencing symptoms of anxiety and depression may be more likely to recall and attribute them to former head injuries (recall bias), especially since neuropsychiatric sequelae of TBI has received considerable media coverage. Alternatively, the association may be a result of common method bias, the correlation of two variables due to a common method of measurement (e.g. self-reported questionnaires), rather than a true association of the underlying constructs.^44^

Our findings are consistent with previous observational studies in retired elite American football and rugby players, which have also found an association between self-reported TBI, depression and sleep,^7-12^ although previous studies have not explored more objective measures of RHI exposure to mitigate the effect of bias. Other studies of people who have been hospitalized with TBI after an accident have found that depression is associated with brain changes, but have not consistently reported any specific anatomical location,^4,15^ and inconsistent alterations in functional connectivity have been observed in areas involved in emotional processing.^16-18^ It is difficult to know whether these findings generalize to RHI and TBI in contact sports due to the differences in mechanisms, and since psychiatric comorbidities are risk factors for TBI in the hospital clinic population.^4^ In this cohort, we found differences between former rugby players and controls in both the mechanism of head injury (sports related versus non-sports related), and in the strength of the association between srTBI and symptoms of depression. Although the association between nBL and symptoms of depression was stronger in former rugby players compared to controls, the association between nDC and symptoms of depression was weaker in former rugby players. Furthermore, we have previously shown that srTBI is associated with self-reported symptoms of depression and sleep dysfunction in the full PREVENT-Dementia cohort (*n* = 617, non-athletes),^45^ but have also found that this association may be related to symptoms of post-traumatic stress disorder following intimate partner violence.^46^ Overall, this suggests that findings from TBI studies in the general population may not necessarily translate to former athletes and vice versa.

Previous neuroimaging studies have found that rugby players have differences in microstructural white matter, and lower volumes of several grey matter regions including the hippocampus and amygdala.^12,22,23^ In this analysis we focused on the hypothalamus, due to its importance in sleep homeostasis, but did not find any group differences between rugby players and controls. However, we did find that more play at the highest level was associated with a lower volume of the anterior-superior hypothalamus. This brain region contains the ventrolateral preoptic and the paraventricular nuclei which are important for regulating sleep and appetite.^47^ Furthermore, this subunit is also affected early in APOE4 homozygotes, who are at high risk of developing Alzheimer’s disease, indicating that this part of the hypothalamus may be particularly susceptible to neurodegeneration.^21^ However, it is not possible to determine from our analysis whether this finding is due to early CTE pathology in the hypothalamus, and the association should therefore be interpreted with caution.

We also found that the former rugby players were significantly more physically active than the controls, even though this was included as a variable in the propensity-matching. This may explain why former rugby players were also less likely to report a previous diagnosis of anxiety and sleep disorders, as exercise is known to reduce depression,^48^ anxiety^49^ and improve sleep quality.^50^ Stigma is an important barrier which stops athletes from seeking support for mental health problems, and this may also have contributed to the lower prevalence of anxiety and sleep disorders in this group.^51^

### Strengths and limitations

A strength of the current analysis is that we used a control group which was matched based on nine risk factors for dementia, several which have also been identified as risk factors for developing depression after TBI in the hospital clinic population.^4^ There were also no group differences in the proxy measures of common sleep problems such as obstructive sleep apnoea, RBD and RLS, which are otherwise prevalent in professional rugby players.^9,11,52-55^

This analysis is based on cross-sectional data which means that associations should not necessarily be interpreted as causative. Most of the retired rugby players in this study have played at national or international level and were recruited via word of mouth, so findings may not generalize to other retired players. For example, it is unclear whether these findings would apply to amateurs, to older players or to those with clinical evidence of cognitive impairment.

We used self-reported questionnaire as the primary measures of TBI exposure, depressive symptoms and sleep, which are inherently prone to both recall bias and common method bias, and may not represent clinically significant depression or sleep dysfunction. Furthermore, it is unclear how well athletic history characteristics such as total career length correlate with RHI exposure, although recent studies have identified player positions which are associated with a higher risk of head impact.^28^ This analysis may also be underpowered to detect significant associations with athletic history characteristics if effect sizes are small (*r* < 0.32). Some studies have argued that self-reported sleep questionnaires (e.g. PSQI) correlate poorly with objective measures of sleep,^56^ although we have previously demonstrated that they can successfully detect differences in sleep with age, sex and depression in medium sized cohort studies.^21^ In addition, the hypothalamic segmentation was acquired with a different version of FreeSurfer compared to other subcortical structures and this may have contributed to the differences from previous findings.

## Conclusions

In this study we analysed cross-sectional data from a cohort of middle-aged, former professional rugby players with a high RHI exposure and srTBI burden. We found that srTBI, but not proxy measures of RHI exposure, was associated with more ongoing symptoms of depression and a higher prevalence of previous depression, anxiety and sleep disorders. We also found that srTBI was indirectly associated with more sleep dysfunction, mediated through increased symptoms of depression. However, the association between srTBI and symptoms of depression was not mediated by differences in grey or white matter structure, indicating that this association may be better explained by psychosocial or methodological factors such as recall bias and common method bias rather than brain injury or early neurodegeneration. We also found that ex-rugby players were less likely to report a previous diagnosis of anxiety or sleep disorders and were no more likely to be depressed, when matched to a control group based on clinical risk factors for dementia and depression. The absence of an association between srTBI measures, symptoms of depression and brain structure should hopefully be reassuring to former rugby players experiencing anxiety, low mood or sleep dysfunction. Considering the recent media coverage on high profile athletes with suspected CTE syndrome, it is natural that many athletes with previous RHI exposure are feeling worried or depressed. Sports associations should focus on reducing the stigma surrounding these symptoms and encourage former players to seek support from healthcare providers. Although we did find that more play at the highest level was associated with a lower volume of the anterior-superior hypothalamus, the clinical relevance of this finding is uncertain. Further studies should further explore the biopsychosocial factors linking RHI exposure and depressive symptoms, and whether this is an early or late symptom of the CTE syndrome.

## Supplementary Material

fcag404_Supplementary_Data

## Data Availability

The PREVENT dataset is available to access through a data request on the study website (https://preventdementia.co.uk/); on the Alzheimer’s Disease Data Initiative (ADDI) platform baseline dataset DOI: https://doi.org/10.34688/PREVENTMAIN_BASELINE_700V1; Dementia Platforms UK (DPUK); and the Global Alzheimer’s Association Network (GAAIN). The code used for this analysis is available here: https://github.com/alaurell/TBI-depression-and-sleep-in-former-rugby-players/

## Appendix

### PREVENT dementia investigators (alphabetical order)

Katie Bridgeman

Leonidas Chouliaras

Siobhan Coleman

Hannah Darwin

Maria-Eleni Dounavi

Feng Deng

Robert Dudas

Sarah Gregory

Ivan Koychev

Brian Lawlor

Audrey Low

Clare Mackay

Elijah Mak

Paresh Malhotra

Graciela Muniz-Terrera

Lorina Naci

John O’Brien

Vanessa Raymont

Craig Ritchie

Li Su

Peter Swann

Tony Thayanandan

Guy Williams
