# Supplementary material for: Traumatic brain injury, symptoms of depression and sleep dysfunction in former elite rugby players
Source: Brain Commun. 2026 Oct 27;8(6):fcag404. doi: 10.1093/braincomms/fcag404 (PMC13739049; doi:10.1093/braincomms/fcag404)
Supplement: Supplementary file 1 [file fcag404_supplementary_data.docx]

**Supplementary results**

Self-reported TBI burden (srTBI) was assessed with three variables: the lifetime number of blows to the head (nBL), the lifetime number of times a head injury resulted in feeling dazed and confused (nDC), and the lifetime number of times a head injury resulted in loss of consciousness (nLC). These were binarised into “high” and “low” exposure groups based on the median values in the rugby group (nBL >10 = high, n=26; nBL ≤10 = low, n=31; nDC >3 = high, n=28; nDC ≤3 = low, n=29; nLC >1 = high, n=26; nLC ≤1 = low, n=31). High nLC (B=3.9[SE:1.3], p(FDR)=0.005) and nBL (B=5.2[SE:1.1], p(FDR)<0.001). but not nDC (B=1.1[SE:1.4], p(FDR)=0.42) were significantly associated with more ongoing symptoms of depression, compared to a low score on these measures. Furthermore, a high nDC was associated with a higher risk of sleep disordered breathing (NoSAS total, B=2.3[SE:0.7], p(FDR)=0.005). None of the binarised measures were significantly associated with hypothalamic volume, hypothalamic subunit volume, Pittsburgh sleep quality index (total and components), Epworth score, suspected rapid eye movement sleep behaviour disorder, suspected restless leg syndrome, or lifetime diagnosis of depression, anxiety or sleep disorder.

-0.20 (-0.47-0.09)

**0.41 (0.27-0.57)**

**0.43 (0.22-0.63)**

Depressive Symptoms

Traumatic Brain Injury (nBL)

Sleep Dysfunction

**Supplementary Figure 1**. There was a strong direct association between a higher lifetime number of blows to the head (nBL) and symptoms of depression (center for epidemiological studies depression scale), and a moderate indirect association between nBL and sleep dysfunction (Pittsburgh sleep quality index), mediated through symptoms of depression. These results were obtained using partial least squares structural equation modelling (n=57).

**0.40 (0.28-0.57)**

0.04 (-0.21-0.29)

**0.30 (0.09-0.46)**

Sup. parietal cortex

Traumatic Brain Injury

Depressive Symptoms

**0.41 (0.25-0.57)**

0.02 (-0.24-0.31)

**0.36 (0.13-0.52)**

Inf. parietal cortex

Traumatic Brain Injury

Depressive Symptoms

**0.39 (0.20-0.57)**

-0.15 (-0.39-0.09)

-0.17 (-0.42-0.15)

Fornix (MD)

Traumatic Brain Injury

Depressive Symptoms

**0.46 (0.33-0.62)**

-0.13 (-0.34-0.05)

**0.29 (0.14-0.45)**

Hippocampal cingulum (FA)

Traumatic Brain Injury

Depressive Symptoms

**0.45 (0.26-0.60)**

**-0.35 (-0.53--0.16)**

0.10 (-0.26-0.39)

Tapetum (FA)

Traumatic Brain Injury

Depressive Symptoms

**0.43 (0.24-0.59)**

**0.24 (0.02-0.47)**

-0.06 (-0.36-0.30)

Tapetum (RD)

Traumatic Brain Injury

Depressive Symptoms

**0.48 (0.30-0.63)**

-0.22 (-0.44-0.04)

**0.31 (0.01-0.52)**

Uncinate fasciculus (AxD)

Traumatic Brain Injury

Depressive Symptoms

**Supplementary Figure 2.** There were 7 brain regions which were significantly associated with either self-reported TBI (srTBI, number of lifetime blows to the head) or symptoms of depression. However, none of these mediated the association between srTBI and symptoms of depression. Solid lines denotes significant associations while dashed lines denotes the non-significant associations between factors. These results were obtained using partial least squares structural equation modelling (n=57). AxD: axial diffusivity; FA: fractional anisotropy; Inf: inferior; MD: mean diffusivity; RD: radial diffusivity; Sup: superior.
